# Advanced Malignant Melanoma: Immunologic and Multimodal Therapeutic Strategies

**DOI:** 10.1155/2010/689893

**Published:** 2010-03-09

**Authors:** Niels Halama, Inka Zoernig, Dirk Jaeger

**Affiliations:** National Center for Tumor Diseases, Department of Medical Oncology, University of Heidelberg, 69120 Heidelberg, Germany

## Abstract

Immunologic treatment strategies are established in malignant melanoma treatment, mainly focusing on Interleukin-2 in advanced disease and interferon alpha in the adjuvant situation. In advanced disease, therapies with IL-2, interferon and different chemotherapeutic agents were not associated with better patient survival in the vast majority of patients. Therefore, an overview of novel immunological agents and combined therapeutic approaches is presented in this review, covering allogenic and autologous vaccine strategies, dendritic cell vaccination, strategies for adoptive immunotherapy and T cell receptor gene transfer, treatment with cytokines and monoclonal antibodies against the CTLA-4 antigen. As emerging treatment strategies are based on individual molecular and immunological characterization of individual tumors/patients, tailored targeted drug therapies move into the focus of treatment strategies. Multimodal combination therapies with considerable potential in altering the immune response in malignant melanoma patients are currently emerging. As oncology moves forward into the field of personalized therapies, a careful molecular and immunological characterization of patients is crucial to select patients for individual targeted treatment.

## 1. Introduction

Surgical removal of tumor tissue is still the most relevant step for the prognosis in treatment of patients with malignant melanomas. In advanced stages of the disease, systemic therapies like chemotherapy are the only relevant treatment options, whereas in the adjuvant situation therapies with interferon alpha are commonly used. Chemotherapy regimens lead to small percentages of objective responses while often causing considerable side effects. Adjuvant treatment with the immunomodulating drug interferon has also significant side effects which are especially burdensome to the patient on long-term treatment regimens. Side effects ranging from myalgias and fever to depression are observed and often cause treatment interruption. Additionally, the improvement of outcome due to interferon therapy is small. In summary, these treatment regimens require the development of novel treatment strategies, especially for the treatment of patients with advanced malignant melanoma. Are we at a standstill or is treatment moving on to new possibilities?

Immunomodulation and direct targeting of signaling pathways in malignant melanoma are promising avenues. Especially the immunogenicity of malignant melanoma tumor cells is important. Spontaneous complete remission can be observed in patients with malignant melanoma and is mainly attributed to the immune response against the tumor. Also an elevated frequency of spontaneous humoral immune responses against tumor antigens was found in melanoma patients. This interaction of the immune system with the tumor shows a promising pathway for intervention and incorporates all portions of the immune system. Novel and contemporary immunomodulatory therapy strategies for malignant melanoma patients are discussed in this review. A key element in these novel strategies is the identification of suitable patients, the selection being based on detailed immunological and molecular characterizations. But the common basis of immunological interventions is to restore tumor-immunity in compromised patients. Tumor-immunity and tumor-tolerance are however intricately interwoven as the evolution of an immune system required the differentiation between “self” and “nonself”. The evolution of the immune system induced the development of a fine tuned balance between “auto-aggressivity” and “tolerance”. Pregnancy is an example for physiological tolerance with multiple regulatory mechanisms involved [1, 2]. In cancer patients, a tumor tolerance is usually found. The therapeutic removal of this tumor-tolerance of course bears the risk of inducing auto-immunity [3]. It is interesting to note, that experiments in mice showed tumor eradication upon depletion of regulatory T cells but also induction of auto-immunity [4]. Another obstacle is the observation that the immunological parameters vary at different time points of a tumor disease. One important hypothesis was introduced by Burnet and Thomas 50 years ago and was termed the “cancer immunosurveillance hypothesis”. This hypothesis proposes an interplay between immune system and tumor cells, whereby the immune system “shapes” tumor cells by eliminating subpopulations of malignant cells. Based on this hypothesis, the concept of “immunoediting” was introduced [5–7]. “Immunoediting” is a dynamic process that has a varying degree of influence on tumor-tolerance and immunogenicity of tumor cells. The reconstruction of the immunologic tumorimmunity is therefore the main aim of current therapeutic immunomodulatory concepts, whether these concepts are based on a single or multiple concomitant therapies.

## 2. Humoral Immune Responses, Tumor Associated Antigens and Monoclonal Antibodies

An interesting aspect of immune response is the presence of spontaneous humoral responses in tumor patients through specific antibodies. Tumor associated antigens (TAA) are molecules that are usually found on tumor cells. Cancer testis antigens represent an important group within these antigens and are mainly found during embryonal development, in testicular tissue and in cancer samples. Spontaneous humoral immune responses against TAA have been reported for different tumor entities [8–14]. For malignant melanoma a prognostic significance of humoral, respective auto-immune, immune responses was found [15]. The significance of this finding and the actual impact of these immune responses are currently debated [16]. 

Another immune intervention is the administration of ex-vivo generated monoclonal antibodies. Therapeutic monoclonal antibodies either act as agonists or antagonists on surface receptors, they can induce apoptosis or they can reduce availability of specific ligands through direct binding. As was seen in clinical trials for the therapeutic antibody cetuximab, antibodies can have multiplying effects even when the target molecule is not present or only in very low concentrations, Antibody dependent cellular cytotoxicity (ADCC) induces cellular immune responses, whereas complement-dependent cytotoxicity (CDC) induces an activation of the complement cascade. Monoclonal antibodies are commonly used in clinical routine for the treatment of multiple tumor entities [17]. 

The hybridoma technology allowed the production of monoclonal mouse antibodies in mice. Mouse antibodies have a clear limitation for clinical application: the induction of allergic reactions. The development of fully humanized antibodies presented a solution to this side effect. These humanized antibodies have a much lower risk of inducing allergic reactions and therefore a vital for patient safety and ensure low toxicity. 

**Figure 1:**
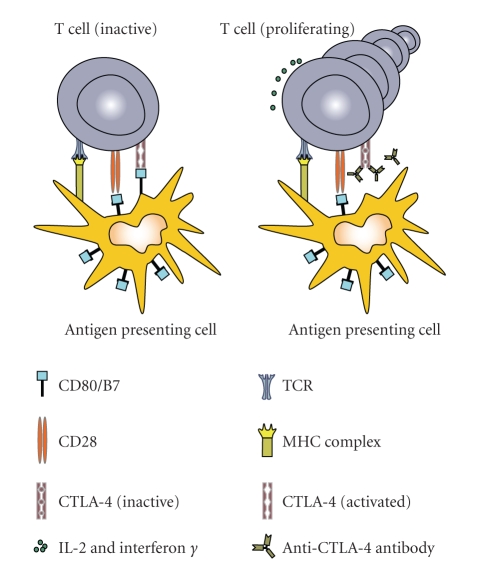
Mechanism of CTLA-4 blockade with inhibitory antibodies. CTLA-4 itself negatively regulates immune responses and blockade leads to increased antigen specific effector T cell responses.

A novel mechanism of antibody treatment is the modulation of the immune system without direct interaction with the tumor. In malignant melanoma patients, two antibodies are being studied with this indirect approach, both targeting “Anti-cytotoxic T lymphocyte-associated antigen 4” (CTLA-4). CTLA-4 receptors in combination with the B7 molecule and the ligand CD28 on T cells are involved in the abrogation of an immune response (see Figure 1), for example, after the successful prevention of an infection. With the interruption of the inhibitory signaling cascade a reversal of peripheral tumor-tolerance can be induced, leading to the induction of an immune response against the tumor [18]. Two humanized monoclonal antibodies against CTLA-4 are currently being investigated in clinical trials: ipilimumab (MDX-010) [19] and tremelimumab (CP-675,206) [20].

Ipilimumab has shown efficacy as single agent and in combination with chemotherapy or vaccination in patients with metastatic malignant melanoma. Similar results were seen for tremelimumab. There was, however, a recent trial that showed no benefit for tremelimumab compared to chemotherapy as first-line treatment [21]. Despite these contradictory observations, the selective inhibition of the CTLA-4 receptor with monoclonal antibodies is seen as an interesting treatment strategy for patients with advanced malignant melanomas. It is here also important to identify predictive marker to identify patients who will benefit from that treatment. An analysis in patients treated with ipilimumab indicated a possible predictive role for the absolute lymphocyte count [22]. Another novel antibody is directed against CD137 (syn. inducible receptor-like protein 4-1BB). CD137 is expressed on CD4 and CD8 positive T cells upon activation and induces further proliferation and activation. Animal models showed an induced anti-tumor immune response after administration of this antibody. Clinical phase I trials were successful and this antibody is currently used in a phase II clinical trial for advanced malignant melanoma patients after conventional pretreatment. 

The immunomodulatory trials pose a new problem with regard to the evaluation of treatment response. Unlike conventional chemotherapy treatment regimens, immunomodulatory drugs or vaccines can show an initial worsening of the clinical situation and following long lasting benefits [23]. This special “bi-phasic” clinical course as a result of immunological interventions is not reflected in current staging protocols or oncological practice. Criteria for the treatment evaluation have to be revised with respect to these observations. Additionally, antibodies can have immune stimulating effects and induce T cell responses or induce further antibody generation, even if the antibody itself does not have a high specificity for the tumor-associated antigen [24, 25]. Furthermore, synergizing effects of immune-based therapy with HER-2/neu targeted vaccination and concomitant administration of an antiangiogenic monoclonal antibody were observed in mouse models [26].

## 3. Vaccination

Vaccination strategies should ideally invoke an effective T cell response in the patient. Existing immunologic tolerance towards the tumor should be broken by this approach. Therefore the careful selection of usable target antigens is important. Optimal target antigens are only expressed homogenously within the tumor. Additionally these target antigens should be common in the respective type of tumor and should have high immunogenicity (see Figure 1). Different useful target antigens were identified [27] and have been used in clinical vaccination studies [12, 28]. The majority of established target antigens belong to the class of “cancer-testis” antigens, which are not expressed in normal tissue except for germ line tissue. These antigens are frequently found in tumor tissue, for example, in multiple myeloma or malignant melanoma. The largest group of possible target antigens belongs however to the “differentiation antigens”. These antigens can be found in a normal differentiated tissue type and in the tumor arising from that tissue type. For example, it can be found in melanocytes and in malignant melanoma cells. 

Vaccines are created in a few ways and vaccination protocols can be based on different strategies. Peptides of the antigen, the whole protein or “naked” DNA can be used for vaccination. The simultaneous use of adjuvants should then enhance the specific T cell response. The selection of the optimal adjuvant substance is still under investigation in studies. All trials published so far have shown very few side effects for vaccination strategies. Hypersensitivity at the injection site and in some cases the induction of autoimmune reactions like vitiligo (upon vaccination with melanocyte differentiation antigens) were seen. So far, no major side effects were seen. Repetitious vaccinations could further enhance specific T cell responses. Pioneering work in this field was done with individually generated autologous vaccines [29]. 64 patients with metastatic malignant melanoma were treated with autologous tumor tissue [30] and showed that clinical and tumor-specific T cell responses could be induced in many patients. A recent study used the autologous vaccine Vitespen (Oncophage). Vitespen is a heatshock-protein-peptide complex [31] that was used in a clinical trial. Results were not as clear cut and therefore it remains unclear whether research with Vitespen is continued. Another vaccination strategy uses dendritic cells. Dendritic cells have a central function in the activation of specific effector T cells. 

On this basis, vaccination strategies with dendritic cells were regarded as a promising therapeutic approach even in advanced tumor diseases [32]. Current data from trials with dendritic cells for patients with malignant melanoma are however not uniform. An enormous problem arises from the variability of protocols in the preparation of dendritic cells and in the vaccination itself. A large phase III trial had to be stopped due to lack of efficacy in the vaccination arm. In this trial peptide-loaded autologous dendritic cells or chemotherapy was given to patients with advanced malignant melanoma [33]. For metastatic malignant melanoma patients peptide-based vaccines have not shown any clinical advantage [34]. Vaccination therapy with little or no side effects is attractive as combination therapy for other modalities like chemotherapy or radiation [35]. But why should a combination be more successful? Data on the immunological effects of chemotherapy or radiation therapy are accumulating [36–38]. During radiation the expression of antigens on the surface of the tumor cells is induced, while a pro-inflammatory microenvironment within the tumor is promoted. So in combination with direct immunological approaches, the combination should lead to greater immunological effects. 

Vaccination strategies have shown variable efficacy in the treatment of solid tumors [39] and appear to be unsuitable for advanced tumor diseases [34]. It has also been proposed, that vaccination strategies can even have detrimental effects for the patients. An important aspect in that discussion seems to be the optimal selection of the target antigen for clinical trials [40]. In adjuvant or minimal residual disease treatment settings, vaccinations have shown benefits for patients. Vaccination with the tumor antigen MAGE-A3 [41] showed for non-small cell lung cancer patients promising results and a subsequent trial was initiated. In malignant melanoma patients, MAGE-A3 protein was tested as first line therapy, showing lasting antibody responses, strong T cell responses and clinical responses [42]. Further trials were initiated and are ongoing. Vaccinations with the cancer testis antigen NY-ESO-1 in “high risk” melanoma patients have also shown encouraging results in a phase II trial. So a clear role for vaccination has yet to be found in the treatment of malignant melanoma patients. From a research perspective, the interplay of antibody presence and the activation of the immune system remain unclear and further investigations will clearly lead to more efficient treatment strategies. Patients at the moment should not be treated with vaccinations outside from clinical trials.

## 4. Interferon and Interleukins

Interferon alpha and interleukin 2 are established immunomodulating substances in the routine treatment of malignant melanoma patients. Objective response rates for interleukin 2 are ~16%, complete remission are seen in ~6% of the cases. Side effects of interleukin-2 treatment are severe and treatment is usually restricted to specialized centers. Interferon is used mainly in the adjuvant situation [43]. Response rates of 10–15% have been observed, in combination with the chemotherapy drug dacarbazine a rate of 24% can be observed. Combination therapies with interleukin 2 and interferon however did not increase the response rate as compared to single agent therapy [44, 45]. The overall survival rates have not increased with the use of interferon and interleukin 2. In search for alternatives, interleukin 21 and 15 are investigated [46, 47]. Structural homologies exist between interleukin 2 and interleukins 21 and 15. The proliferation of regulatory T cells is not induced by the latter two interleukins. As recent studies elucidated the intratumoral cytokine milieu, the debate on useful cytokine (or chemokine) administration in malignant melanoma patients is likely to increase [48].

## 5. Adoptive Immune Cell Strategies

**Figure 2:**
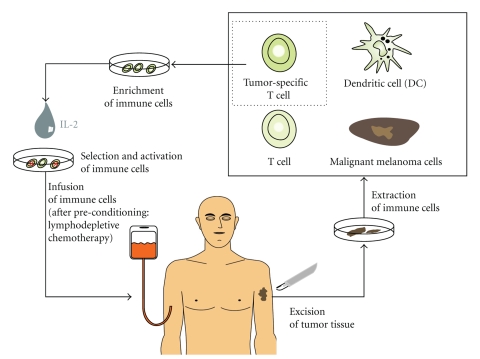
Principle of adoptive immunotherapy.

One way to circumvent the established tumor tolerance in tumor patients is to administer exogenous antibodies. A similar approach on T cell level is termed “adoptive immunotherapy”. Immune cells of the patient are activated or primed outside of the patient and are then given back. This intervention with ex vivo stimulation or modification of immune cells tries to avoid the immunosuppression or tumortolerance in the patient. There are different variants of adoptive immunotherapies (see Figure 2). One possible application is the expansion of autologous tumor-reactive T cells, the activation and subsequent re-infusion. A protocol with extraction of tumor-infiltrating T cells and pre-conditioning of the patient before re-infusion with a lymphodepletive chemotherapy showed response rates of around 50% [49, 50]. These results not only indicated a possible inhibitory role of the immune system-respective the tumor environment but also highlighted the immunomodulatory effects of chemotherapy. Inhibitory immune cells are thought to play a key role in the chemotherapy-induced alterations in the local tumor environment. Myeloid-derived suppressor cells (MDSCs) and regulatory T cells influence the immune response [4, 51]. The above mentioned study has demonstrated the feasibility of this combined approach, some of the patients had long lasting antigen-specific T cells, long after the re-infusion of the expanded T cells. The population of expanded T cells was derived from the tumor, tumor-infiltrating lymphocytes (TILs). Earlier approaches with adoptive immunotherapies using circulating lymphocytes (lymphokine-activated killer cells, LAKs) did not yield good clinical results. Difficulties with this concept can arise in tumors where it is difficult to extract lymphocytes. Immune cell transfers can of course facilitate other immune cell populations, like natural killer cells [52].

## 6. T-Cell Receptor Transfer

**Figure 3:**
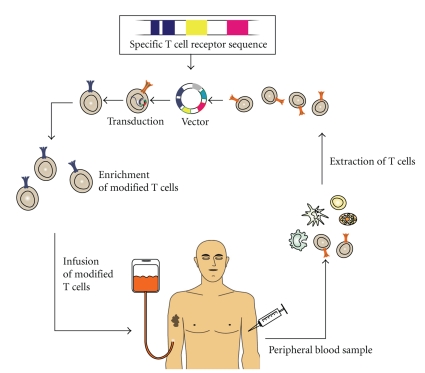
T cell receptor transfer.

Another way of inducing tumor-specific immune responses is the direct transfer of tumor-specificity to non-tumor-specific T cells. The principle of “T cell receptor transfer” is the retroviral transfer of specific alpha- and beta-chains of a specific T cell receptor (TCR) (see Figure 3) [53]. Retroviral transfer of genetic information has risks because with stable integration errors can occur, possibly resulting in unwanted effects. One way to ensure safety is to use “self-destructive” vectors. Another problem that can arise with T cell receptor transfer is the generation of chimeric T cell receptors. Chimeric receptors have endogenous alpha- or beta-chains mixed with the modified or transfected ones. This can lead to unforeseen T cell specificities and therefore lead to incalculable side effects. A solution to this problem is the use of “hybrid T cell receptors” [54]. These T cell receptors contain murine constant regions and human variable regions. This leads to a majority of correct pairings and leads to other improved immunological properties.

## 7. Targeted Pathway Inhibition

Why a section on “targeted pathway inhibition” in a review on immunological and multimodal treatment strategies in malignant melanoma patients? Targeted agents can alter the proteins involved in key pathways, such as apoptotic pathways and thereby improve, for example immunotherapies. The detailed investigations on signaling pathways in tumor cells lead to specific drugs for selective inhibition. The direct inhibition of signaling pathways has already lead to enormous improvements in other tumor entities [55, 56]. In the analyses of the effects of pathway inhibition data is accumulating that shows an effect of this inhibition on antigen presentation and tumor cell recognition [57, 58]. In malignant melanomas multiple different aberations in different pathways are found. Upregulation of the RAS/RAF/MEK signaling pathway [59, 60], an upregulation of the PI3K/Akt3 pathway via a mutation of the PTEN gene [61], a decreased expression of the retinoblastoma protein (RB) via cyclin D1 or CDK4, activating c-Kit mutations and inactivation of the CDKN2A gene can often be found in melanomas. Small molecule inhibitors like sorafenib inhibit multiple kinases, for example, B-RAF, C-RAF, VEGFR-2, PDGFR and c-KIT, and sorafenib has shown efficacy as single agent and in combination with chemotherapy in patients with advanced malignant melanoma. The inhibition of the RAS/RAF/MEK/ERK pathway (and others like the Akt, PI3K, mTOR or hedghehog pathway) has more beneficial effects for immunological treatments, as the expression of anti-apoptotic proteins are downregulated. Effective T cell responses against tumor cells induce tumor cell apoptosis. Tumor cells often develop strategies to inhibit induced apoptosis, so that targeting of anti-apoptotic proteins in the treatment of malignant melanoma patients can strengthen the effectiveness of the immune response [62]. The full range of immunological effects of targeted therapies has to be investigated in future research to fully harness the potential of these treatments.

## 8. Conclusion

In summary, melanoma is a unique cancer because advanced disease can respond to immune surveillance. Working from the principles of spontaneous regression and unique tumor antigens such as the testis antigen, several principles of immune function are now understood. In addition to the established, albeit toxic and rather ineffective treatments of interferon alpha in the adjuvant setting and Interleukin 2 in the advanced setting, new therapies with anti-CTLA-4 antibodies or specific (individualized) tumor vaccines are being developed. Adoptive transfer with tumor infiltrating lymphocytes may hold promise. Melanoma pathways are better understood and targeted therapies to the RAF/RAS/MEK/ERK or other signaling pathways could be combined with immune treatments. The existing results are promising and new avenues will open with a detailed understanding of the immunological interactions of tumor and immune cells and the immunological effects of modern (targeted) drug therapies.

## References

[1] Mold JE, Michaelsson J, Burt TD (2008). Maternal alloantigens promote the development of tolerogenic fetal regulatory T cells in utero. *Science*.

[2] Sharkey AM, Gardner L, Hiby S, Farrell L (2008). Killer Ig-like receptor expression in uterine NK cells is biased toward recognition of HLA-C and alters with gestational age. *Journal of Immunology*.

[3] Ohnmacht C, Pullner A, King SBS (2009). Constitutive ablation of dendritic cells breaks self-tolerance of CD4 T cells and results in spontaneous fatal autoimmunity. *Journal of Experimental Medicine*.

[4] Shimizu J, Yamazaki S, Sakaguchi S (1999). Induction of tumor immunity by removing CD25^+^CD4^+^ T cells: a common basis between tumor immunity and autoimmunity. *Journal of Immunology*.

[5] Bui JD, Schreiber RD (2007). Cancer immunosurveillance, immunoediting and inflammation: independent or interdependent processes?. *Current Opinion in Immunology*.

[6] Dunn GP, Bruce AT, Ikeda H, Old LJ, Schreiber RD (2002). Cancer immunoediting: from immunosurveillance to tumor escape. *Nature Immunology*.

[7] Koebel CM, Vermi W, Swann JB (2007). Adaptive immunity maintains occult cancer in an equilibrium state. *Nature*.

[8] Jager E, Chen Y-T, Drijfhout JW (1998). Simultaneous humoral and cellular immune response against cancer-testis antigen NY-ESO-1: definition of human histocompatibility leukocyte antigen (HLA)-A2-binding peptide epitopes. *Journal of Experimental Medicine*.

[9] Jager E, Gnjatic S, Nagata Y (2000). Induction of primary NY-ESO-1 immunity: CD8+ T lymphocyte and antibody responses in peptide-vaccinated patients with NY-ESO-1+ cancers. *Proceedings of the National Academy of Sciences of the United States of America*.

[10] Jager E, Jager D, Knuth A (2003). Antigen-specific immunotherapy and cancer vaccines. *International Journal of Cancer*.

[11] Jager D, Karbach J, Pauligk C (2005). Humoral and cellular immune responses against the breast cancer antigen NY-BR-1: definition of two HLA-A2 restricted peptide epitopes. *Cancer Immunity*.

[12] Jager D, Stockert E, Gure AO (2001). Identification of a tissue-specific putative transcription factor in breast tissue by serological screening of a breast cancer library. *Cancer Research*.

[13] Jager D, Filonenko V, Gout I (2007). NY-BR-1 is a differentiation antigen of the mammary gland. *Applied Immunohistochemistry and Molecular Morphology*.

[14] Scanlan MJ, Welt S, Gordon CM (2002). Cancer-related serological recognition of human colon cancer: identification of potential diagnostic and immunotherapeutic targets. *Cancer Research*.

[15] Gogas H, Ioannovich J, Dafni U (2006). Prognostic significance of autoimmunity during treatment of melanoma with interferon. *The New England Journal of Medicine*.

[16] Bouwhuis MG, Suciu S, Collette S (2009). Autoimmune antibodies and recurrence-free interval in melanoma patients treated with adjuvant interferon. *Journal of the National Cancer Institute*.

[17] Adams GP, Weiner LM (2005). Monoclonal antibody therapy of cancer. *Nature Biotechnology*.

[18] Melero I, Hervas-Stubbs S, Glennie M, Pardoll DM, Chen L (2007). Immunostimulatory monoclonal antibodies for cancer therapy. *Nature Reviews Cancer*.

[19] Weber JS, O’Day S, Urba W (2008). Phase I/II study of ipilimumab for patients with metastatic melanoma. *Journal of Clinical Oncology*.

[20] Ribas A, Hanson DC, Noe DA (2007). Tremelimumab (CP-675,206), a cytotoxic T lymphocyte-associated antigen 4 blocking monoclonal antibody in clinical development for patients with cancer. *Oncologist*.

[21] Ribas A, Hauschild A, Kefford R (2008). Phase III, open-label, randomized, comparative study of tremelimumab CP-675,206 and chemotherapy temozolomide [TMZ] or dacarbazine [DTIC] in patients with advanced melanoma. *Journal of Clinical Oncology*.

[22] Weber J, Thompson JA, Hamid O (2009). A randomized, double-blind, placebo-controlled, phase II study comparing the tolerability and efficacy of ipilimumab administered with or without prophylactic budesonide in patients with unresectable stage III or IV melanoma. *Clinical Cancer Research*.

[23] Saenger YM, Wolchok JD (2008). The heterogeneity of the kinetics of response to ipilimumab in metastatic melanoma: patient cases. *Cancer Immunity*.

[24] Harbers SO, Crocker A, Catalano G (2007). Antibody-enhanced cross-presentation of self antigen breaks T cell tolerance. *Journal of Clinical Investigation*.

[25] Taylor C, Hershman D, Shah N (2007). Augmented HER-2-specific immunity during treatment with trastuzumab and chemotherapy. *Clinical Cancer Research*.

[26] Manning EA, Ullman JGM, Leatherman JM (2007). A vascular endothelial growth factor receptor-2 inhibitor enhances antitumor immunity through an immune-based mechanism. *Clinical Cancer Research*.

[27] Scanlan MJ (2005). Identification of human tumor antigens by serological analysis of recombinant cDNA expression libraries (SEREX). *Current Protocols in Immunology*.

[28] Yokoe T, Tanaka F, Mimori K (2008). Efficient identification of a novel cancer/testis antigen for immunotherapy using three-step microarray analysis. *Cancer Research*.

[29] Srivastava P (2002). Interaction of heat shock proteins with peptides and antigen presenting cells: chaperoning of the innate and adaptive immune responses. *Annual Review of Immunology*.

[30] Belli F, Testori A, Rivoltini L (2002). Vaccination of metastatic melanoma patients with autologous tumor-derived heat shock protein gp96-peptide complexes: clinical and immunologic findings. *Journal of Clinical Oncology*.

[31] Testori A, Richards J, Whitman E (2008). Phase III comparison of vitespen, an autologous tumor-derived heat shock protein gp96 peptide complex vaccine, with physician’s choice of treatment for stage IV melanoma: the C-100-21 study group. *Journal of Clinical Oncology*.

[32] Banchereau J, Palucka AK (2005). Dendritic cells as therapeutic vaccines against cancer. *Nature Reviews Immunology*.

[33] Schadendorf D, Ugurel S, Schuler-Thurner B (2006). Dacarbazine (DTIC) versus vaccination with autologous peptide-pulsed dendritic cells (DC) in first-line treatment of patients with metastatic melanoma: a randomized phase III trial of the DC study group of the DeCOG. *Annals of Oncology*.

[34] Rosenberg SA, Yang JC, Restifo NP (2004). Cancer immunotherapy: moving beyond current vaccines. *Nature Medicine*.

[35] Stewart IV JH, Rosenberg SA (2000). Long-term survival of anti-tumor lymphocytes generated by vaccination of patients with melanoma with a peptide vaccine. *Journal of Immunotherapy*.

[36] Kaneno R, Shurin GV, Tourkova IL, Shurin MR (2009). Chemomodulation of human dendritic cell function by antineoplastic agents in low noncytotoxic concentrations. *Journal of Translational Medicine*.

[37] North RJ (1982). Cyclophosphamide-facilitated adoptive immunotherapy of an established tumor depends on elimination of tumor-induced suppressor T cells. *Journal of Experimental Medicine*.

[38] Shurin GV, Tourkova IL, Kaneno R, Shurin MR (2009). Chemotherapeutic agents in noncytotoxic concentrations increase antigen presentation by dendritic cells via an IL-12-dependent mechanism. *Journal of Immunology*.

[39] Eggermont AMM (2009). Immunotherapy: vaccine trials in melanoma—time for reflection. *Nature Reviews Clinical Oncology*.

[40] Buckwalter MR, Srivastava PK (2008). “It is the antigen(s), stupid” and other lessons from over a decade of vaccitherapy of human cancer. *Seminars in Immunology*.

[41] Atanackovic D, Altorki NK, Cao Y (2008). Booster vaccination of cancer patients with MAGE-A3 protein reveals long-term immunological memory or tolerance depending on priming. *Proceedings of the National Academy of Sciences of the United States of America*.

[42] Kruit W, Suciu S, Dreno B (2008). Immunization with recombinant MAGE-A3 protein combined with adjuvant systems AS15 or AS02B in patients with unresectable and progressive metastatic cutaneous melanoma: a randomized open-label phase II study of the EORTC Melanoma Group (16032–18031). *Journal of Clinical Oncology*.

[43] Hauschild A, Weichenthal M, Rass K (2009). Prospective randomized multicenter adjuvant dermatologic cooperative oncology group trial of low-dose interferon alfa-2b with or without a modified high-dose interferon alfa-2b induction phase in patients with lymph node—negative melanoma. *Journal of Clinical Oncology*.

[44] Sparano JA, Fisher RI, Sunderland M (1993). Randomized phase III trial of treatment with high-dose interleukin-2 either alone or in combination with interferon alfa-2a in patients with advanced melanoma. *Journal of Clinical Oncology*.

[45] Marincola FM, White DE, Wise AP, Rosenberg SA (1995). Combination therapy with interferon alfa-2a and interleukin-2 for the treatment of metastatic cancer. *Journal of Clinical Oncology*.

[46] Frederiksen KS, Lundsgaard D, Freeman JA (2008). IL-21 induces in vivo immune activation of NK cells and CD8^+^ T cells in patients with metastatic melanoma and renal cell carcinoma. *Cancer Immunology, Immunotherapy*.

[47] Thompson JA, Curti BD, Redman BG (2008). Phase I study of recombinant interleukin-21 in patients with metastatic melanoma and renal cell carcinoma. *Journal of Clinical Oncology*.

[48] Harlin H, Meng Y, Peterson AC (2009). Chemokine expression in melanoma metastases associated with CD8^+^
T-cell recruitment. *Cancer Research*.

[49] Dudley ME, Wunderlich JR, Yang JC (2005). Adoptive cell transfer therapy following non-myeloablative but lymphodepleting chemotherapy for the treatment of patients with refractory metastatic melanoma. *Journal of Clinical Oncology*.

[50] Dudley ME, Yang JC, Sherry R (2008). Adoptive cell therapy for patients with metastatic melanoma: evaluation of intensive myeloablative chemoradiation preparative regimens. *Journal of Clinical Oncology*.

[51] Zou W (2006). Regulatory T cells, tumour immunity and immunotherapy. *Nature Reviews Immunology*.

[52] Pegram HJ, Jackson JT, Smyth MJ, Kershaw MH, Darcy PK (2008). Adoptive transfer of gene-modified primary NK cells can specifically inhibit tumor progression in vivo. *Journal of Immunology*.

[53] Stauss HJ, Cesco-Gaspere M, Thomas S (2007). Monoclonal t-cell receptors: new reagents for cancer therapy. *Molecular Therapy*.

[54] Voss R, Willemsen RA, Kuball J (2008). Molecular design of the C*α*
*β* interface favors specific pairing of introduced TCR*α*
*β* in human T cells. *Journal of Immunology*.

[55] Abou-Alfa GK, Schwartz L, Ricci S (2006). Phase II study of sorafenib in patients with advanced hepatocellular carcinoma. *Journal of Clinical Oncology*.

[56] Seront E, Machiels J-P (2009). Targeted therapies in the treatment of advanced renal cell carcinoma. *Recent Patents on Anti-Cancer Drug Discovery*.

[57] Sers C, Kuner R, Falk CS (2009). Down-regulation of HLA class I and NKG2D ligands through a concerted action of MAPK and DNA methyltransferases in colorectal cancer cells. *International Journal of Cancer*.

[58] Preta G, Marescotti D, Fortini C (2008). Inhibition of serine-peptidase activity enhances the generation of a survivin-derived HLA-A2-presented CTL epitope in colon-carcinoma cells. *Scandinavian Journal of Immunology*.

[59] Meier F, Schittek B, Busch S (2005). The Ras/Raf/MEK/ERK and PI3K/AKT signaling pathways present molecular targets for the effective treatment of advanced melanoma. *Frontiers in Bioscience*.

[60] Curtin JA, Fridlyand J, Kageshita T (2005). Distinct sets of genetic alterations in melanoma. *The New England Journal of Medicine*.

[61] LoPiccolo J, Blumenthal GM, Bernstein WB, Dennis PA (2008). Targeting the PI3K/Akt/mTOR pathway: effective combinations and clinical considerations. *Drug Resistance Updates*.

[62] Hersey P, Zhang XD (2009). Treatment combinations targeting apoptosis to improve immunotherapy of melanoma. *Cancer Immunology, Immunotherapy*.

